# Reproductive Risk Factor Patterns in Caribbean Women With Breast Cancer Across 4 Generations

**DOI:** 10.1001/jamanetworkopen.2024.38091

**Published:** 2024-10-08

**Authors:** Alex P. Sanchez-Covarrubias, Maurice J. Chery, Priscilla Barreto-Coehlo, Cheryl Alexis, Jameel Ali, Alexandra Diaz-Barbe, Raleigh Butler, Saida Bowe, DuVaughn Curling, Vincent DeGennaro, Leah V. Dodds, Hedda Dyer, Darron Halliday, Patricia Jeudin, Dwight Lowe, Kristy Samaroo, Gillian Wharfe, Matthew Schlumbrecht, Isildinha M. Reis, Judith Hurley, Sophia George

**Affiliations:** 1 Cancer Biology Graduate Program, Miller School of Medicine, University of Miami, Miami, Florida; 2 Division of Gynecologic Oncology, Department of Obstetrics, Gynecology, and Reproductive Sciences, Miller School of Medicine, University of Miami, Miami, Florida; 3 Sylvester Comprehensive Cancer Center, Miami, Florida; 4 Division of Prevention Science, Department of Public Health Sciences, Miller School of Medicine, University of Miami, Miami, Florida; 5 Division of Medical Oncology, Department of Medicine, Miller School of Medicine, University of Miami, Miami, Florida; 6 University of the West Indies–Cave Hill, Bridgetown, Barbados; 7 St James Medical Complex, North Northwest Regional Health Authority, Port-of-Spain, Trinidad and Tobago; 8 Department of Obstetrics and Gynecology, School of Clinical Medicine and Research, Princess Margaret Hospital, University of the West Indies, Nassau, The Bahamas; 9 Innovating Health International, Port au Prince, Haiti; 10 Ross University School of Medicine, St Michael, Barbados; 11 University of the West Indies–Mona, Kingston, Jamaica; 12 Cayman Islands Cancer Society, Grand Cayman, Cayman Islands; 13 Division of Statistics, Department of Public Health Sciences, Miller School of Medicine, University of Miami, Miami, Florida

## Abstract

**Importance:**

Breast cancer (BC) is commonly diagnosed among Caribbean women. Shifts in reproductive patterns modify the incidence of BC diagnosis and age at BC diagnosis in population-based studies; however, reproductive patterns in Caribbean women remain understudied.

**Objective:**

To describe the temporal trends in reproductive patterns and age at BC diagnosis in Caribbean-born women.

**Design, Setting, and Participants:**

A cross-sectional observational study—the Caribbean Women’s Cancer Study—was conducted, with data on reproductive patterns known to affect BC risk collected in The Bahamas, Barbados, Cayman Islands, Dominica, Haiti, Jamaica, and Trinidad and Tobago. Participants were recruited prospectively. The sample included women born in Caribbean countries and diagnosed with invasive BC and/or ovarian cancer from June 1, 2010, to June 30, 2018, and was divided into 4 birth cohorts (born before 1950, 1950-1959, 1960-1969, and in or after 1970). Data were analyzed between August 1, 2023, and July 31, 2024.

**Exposures:**

Receipt of a BC diagnosis and birth in a Caribbean country.

**Main Outcomes and Measures:**

Change in reproductive patterns between birth cohorts, including age at BC diagnosis, family history of cancer, age at first pregnancy, number of pregnancies, number of full-term pregnancies, number of siblings, age at menarche and menopause, estrogen receptor status, and germline pathogenic/likely pathogenic variants.

**Results:**

Of 1015 participants diagnosed with BC and ovarian cancer, 995 women (mean [SD] age, 46.6 [10.8] years; 605 [81.8%] Afro-Caribbean, 98 [13.2%] East Indian, 22 [3.0%] White, and 12 [1.6%] >1 race) received a diagnosis of invasive BC. Comparison from older to younger birth cohorts (presented in the order of born before 1950, 1950-1959, 1960-1969 and in or after 1970) showed an increased proportion of women experiencing menarche at age 12 years or younger (33.0% vs 47.3% vs 45.5% vs 57.9%; *P* < .001), women with no pregnancies (6.8% vs 6.8% vs 10.5% vs 22.8%; *P* < .001), and nulliparous women (8.6% vs 9.2% vs 13.9% vs 27.6%; *P* < .001). Younger age at BC diagnosis was observed in women experiencing menarche at age 12 years or younger (mean [SD], 45.0 [10.5] years) vs 15 years or older (mean [SD], 49.1 [11.2] years) and in nulliparous women (mean [SD], 42.1 [11.2] years) vs 3 or more full-term pregnancies (mean [SD], 49.9 [10.6] years; *P* < .001). For every year of first pregnancy delay, women had a 4% increased chance of being diagnosed with estrogen receptor–positive tumors (odds ratio, 1.04; 95% CI, 1.01-1.08; *P* = .02).

**Conclusions and Relevance:**

In this cross-sectional study, between each 10-year birth cohort, women diagnosed with BC had a lower age at menarche, number of pregnancies, and number of full-term pregnancies. These findings suggest that interventions targeting other BC risk factors need to be implemented.

## Key Points

**Question:**

Have reproductive patterns changed within generations of women diagnosed with breast cancer in Caribbean countries?

**Findings:**

In this cross-sectional study including 995 women diagnosed with breast cancer in 7 countries in the Caribbean, reproductive patterns shifted. Within four 10-year generations, significant decreases were observed in the mean number of siblings, age at menarche, number of pregnancies, and number of full-term pregnancies.

**Meaning:**

This study observed changes in reproductive patterns in women with breast cancer in the Caribbean region, suggesting that interventions targeting other breast cancer risk factors need to be implemented.

## Teaser

This cross-sectional study examines changes over 4 decades in factors related to reproduction in women in Caribbean countries with a diagnosis of invasive breast cancer.

## Introduction

Breast cancer (BC) accounts for the largest proportion of all cancer cases and is the fourth leading cause of cancer mortality worldwide.^1^ Although the incidence of BC is 88% higher in high-income countries, women living in low- to middle-income countries have 17% higher mortality rates.^1^ The Caribbean region has one of the highest BC mortality rates, with The Bahamas, Barbados, and Jamaica experiencing the world’s highest mortality.^2,3^ In the Caribbean, BC is prevalent among premenopausal women and is commonly diagnosed at a younger age (mean age: The Bahamas, 56.6; Barbados, 57.9; Haiti, 49; Jamaica, 49; and Trinidad and Tobago, 43 years)^4,5,6,7^ compared with the US (median age, 63 years).^8^

Several factors can explain the disproportionate burden of BC diagnoses in the Caribbean, such as obesity,^9,10^ genetics, parity, age at menarche, age at first pregnancy, and family size.^11,12,13^ These patterns differ between countries, oftentimes reflecting varying levels of economic development. The intersection of socioeconomic growth and changing lifestyle patterns plays a major role in shifting health outcomes. As low- to middle-income countries undergo economic transitions toward a higher development index, their populations tend to adopt a more sedentary lifestyle, gain weight, and display increased smoking and alcohol intake.^14,15^ Reproductive patterns also lean toward modern lifestyle norms, characterized by delayed first childbirth and fewer overall pregnancies.^16,17^

In a previous study examining the prevalence of hereditary breast and ovarian cancer syndrome (HBOC) in the Caribbean, individuals from The Bahamas had the highest prevalence of HBOC.^18^ Furthermore, 80% of Caribbean women diagnosed with BC and carrying a pathogenic/likely pathogenic germline variant had a relative diagnosed with BC.^19^ This initial Caribbean-based cross-sectional study observed early onset and highly aggressive disease in women with HBOC.^19^

The rich cultural and ethnic diversity of the Caribbean region also provides a multifaceted lens to examine these health outcomes. The Caribbean population is predominantly of African ancestry but also includes Asian, East Indian, European, Indigenous, and Middle Eastern ancestries.^18^ This admixture of genomic ancestries potentially affects the disease patterns we observe.

The interplay of genetic variants, genomic ancestries, and reproductive patterns within this uniquely diverse population is a largely understudied area, representing a major gap in the literature that our study aims to address. Considering the high rates of BC in the Caribbean, we sought to examine reproductive patterns across 7 countries in the region.

## Methods

### Cohort and Eligibility Criteria

This cross-sectional study is a subset of the Caribbean Women’s Cancer Study (CWCS), which prospectively enrolled patients with invasive BC and/or ovarian cancer born in Caribbean countries from June 1, 2010, to June 30, 2018. Details of the CWCS protocol approval were published previously.^18^ From the CWCS we selected participants who self-identified as female and received a diagnosis of invasive primary BC at any age. The study was approved by the institutional review boards at the Ministries of Health in The Bahamas, Barbados, Cayman Islands, Dominica, and Haiti; and at the University of West Indies, Mona, Jamaica; the Northwest Regional Health Authority of Trinidad and Tobago; and the University of Miami. Participants provided written informed consent; no financial compensation was given. The study followed the Strengthening the Reporting of Observational Studies in Epidemiology (STROBE) reporting guideline.

### Data Reporting

Eligible women were divided into 4 birth cohorts: before 1950, between 1950 and 1959, between 1960 and 1969, and in or after 1970. The following variables were collected from the participants through a survey: family history of BC and ovarian cancer, age at first pregnancy, number of pregnancies, number of full-term pregnancies (FTPs), number of biologic siblings, age at menarche, menopausal status, and age at menopause. For race and ethnicity identification, participants chose among 12 categories; selecting multiple options was allowed. If the option Other was selected, they could write how they self-identified. For analysis purposes, categories were combined as Asian (includes Chinese or Other part of Asia), Black or Afro-Caribbean (includes African American, Afro-Caribbean, and Black) East Indian (includes India), 2 or more races (includes when participants selected ≥2 or more categories [eg, Chinese or other part of Asia, Afro-Caribbean, and White/Caucasian]), White or Caucasian (includes White/Caucasian and European).

Body mass index (BMI) at the time of diagnosis, age at BC diagnosis, and total abdominal hysterectomy with salpingo-oophorectomy (TAH-BSO) status were abstracted from the medical records. Estrogen receptor (ER) was measured by immunohistochemistry when available and BC was classified as ER-positive (≥1%) or ER negative (<1%) as determined by a local pathologist. Age at first pregnancy was reported in a subset of women with at least 1 FTP (parous). Age at natural menopause was the age at menopause reported in a subset of women who were postmenopausal and did not undergo TAH-BSO. Women underwent genetic testing as described previously^18^; for germline variant status, benign variants and variants of unknown significance were grouped as negative germline pathogenic/likely pathogenic variant, whereas likely pathogenic and pathogenic variants were characterized as positive germline pathogenic/likely pathogenic variant.^18^

The term *fertility* is used herein when referring to total reproductive experience, without accounting for its physiologic or social determinants, which is consistent with use of the term in other studies associating BC risk with fertility.^20^ Age at menarche was categorized as age 12 or less, 13, 14, and 15 years or older; number of pregnancies as 0, 1, 2, and 3 or more; number of FTP as 0 (nulliparous), 1, 2, and 3 or more; age at natural menopause as age 45 or younger, 46 to 50, and older than 50 years; age at first pregnancy as less than 20, 20 to 24, 25 to 29, and 30 years or older; and number of siblings as none, 1 to 5, and more than 5. These variables were also reported as means. Age at BC diagnosis was reported as a continuous variable. Body mass index was classified as less than 25.0, 25.0 to 29.9, and 30.0 or greater (calculated as weight in kilograms divided by height in meters squared). Family history of BC or ovarian cancer and germline pathogenic/likely pathogenic variant status were dichotomous (yes/positive or no/negative). We analyzed associations of reproductive factors with age at BC diagnosis using the defined categories. We assessed associations between age at BC diagnosis, germline variant status, ER-positive BC, and reproductive patterns.

### Statistical Analysis

Descriptive statistics were used to describe the 4 birth cohorts. Continuous variables were compared using a 2-tailed independent *t* test or 1-way analysis of variance when appropriate. Categorical variables were compared using the χ^2^ test or Fisher exact test. Missing data accounted for less than 10% of the data; therefore, missing data were excluded in analyses and the total number of observations used in the analysis is reported in each table. Correlation between age at BC diagnosis and other continuous variables was explored using the Spearman rank correlation coefficient (ρ). Logistic regression tested reproductive factors as estimators for the likelihood of positive germline variant or ER-positive BC. All tests were 2-tailed, and *P* < .05 was considered statistically significant. Data analysis was conducted using SAS software, version 9.4 (SAS Institute Inc) and R, version 4.3.2 (R Foundation for Statistical Computing). Data were analyzed between August 1, 2023, and July 31, 2024.

## Results

From the 1015 women who were enrolled in the CWCS, the analytical sample included 995 self-identified women who had a primary diagnosis of BC. Mean (SD) age at BC diagnosis was 46.6 (10.8) years; 3 (0.4%) women self-identified as Asian, 605 (81.8%) as Black or Afro-Caribbean, 98 (13.2%) as East Indian, 22 (3.0%) as White, and 12 (1.6%) as 2 or more races. Most women were premenopausal (558 [57.5%]), had a BMI greater than or equal to 30 (399 [41.5%]), experienced menarche before age 12 years (453 [46.7%]), reported 3 or more FTPs (404 [41.1%]), and experienced pregnancy between ages 20 and 24 years (289 [35.1%]); further demographic information is reported in Table 1. The 4 birth cohorts included 177 women born before 1950, 254 born between 1950 and 1959, 330 between 1960 and 1969, and 234 born in or after 1970. Excluding the cohort born in or after 1970, the mean (SD) age at BC diagnosis decreased steadily with each successive decade of birth from before 1950, 1950 to 1959, and 1960 to 1969 (60.7 [9.4] vs 50.4 [7.6] vs 44.3 [4.9]; *P* < .001). Women born between 1960 and 1969 received a BC diagnosis at a younger mean age (44.3 years) compared with women born between 1950 and 1959 (50.4 years) and women born before 1950 (60.7 years). For each of the 4 birth groups, most participants had overweight (BMI 25.0-29.9) or obesity (BMI ≥30). The proportion of women with obesity was lowest among those born in or after 1970 (34.9%) compared with those born before 1950 (45.4%), 1950 to 1959 (45.3%) and 1960 to 1969 (41.1 %) (*P* = .02); however, stratification of BMI by menopausal status revealed no association between BMI and birth groups (eTable 1 in Supplement 1). There was no association across successive decade of birth in the order of born before 1950, 1950-1959, 1960-1969 and in or after 1970 for positive family history of BC (52.5% vs 54.6% vs 49.7% vs 45.3%; *P* = .02) or ovarian cancer (8.6% vs 10.5% vs 12.0% vs 11.2%; *P* = .71). Across the 4 birth cohorts (born before 1950, 1950-1959, 1960-1969 and in or after 1970), there were significant differences in the means of the number of biological siblings (6.6 vs 5.8 vs 6.1 vs 4.5; *P* < .001), suggesting a transition from larger to smaller families. Other notable differences included an increased proportion of women experiencing menarche at or before age 12 years (33.0% vs 47.3% vs 45.5% vs 57.9%; *P* < .001), an increased proportion of no pregnancies (6.8% vs 6.8% vs 10.5% vs 22.8%; *P* < .001), an increased proportion of nulliparous women (8.6% vs 9.2% vs 13.9% vs 27.6%; *P* < .001), and an increased proportion of women undergoing natural menopause at or before age 45 years (22.7% vs 30.6% vs 59.6%; *P* < .001 [group born in or after 1970 excluded]) in women born in later decades. Fewer women underwent TAH-BSO in younger birth groups (32% vs 20.8% vs 13.2% vs 5.1%; *P*<.001). There was an increasing proportion of BCs associated with positive germline variants over decades (9.6% vs 11.8% vs 15.8% vs 17.1%) and ER-negative BC (32.1% vs 37.6% vs 42.6% vs 43.9%), although the finding was not statistically significant. These results are summarized in Table 2.

**Table 1.** Demographic Characteristics of the Entire Study Population

Variable	Participants, No. (%)
Age at BC diagnosis, mean (SD), y	46.6 (10.8)
Self-identified race^a^	
Asian	3 (0.4)
Black or Afro-Caribbean	605 (81.8)
East Indian	98 (13.2)
≥2 Races	12 (1.6)
White or Caucasian	22 (3.0)
Unknown/missing	250
BMI	
<25.0	243 (25.3)
25.0-29.9	320 (33.3)
≥30.0	399 (41.5)
Unknown/missing	66
Family history of breast cancer	
Yes	497 (50.4)
No	489 (49.6)
Unknown/missing	18
Family history of ovarian cancer	
Yes	106 (10.8)
No	874 (89.2)
Unknown/missing	30
No. of siblings	
Mean (SD)	5.8 (4.1)
0	24 (2.9)
1-5	411 (50.3)
>5	382 (46.8)
Unknown/missing	178
Age at menarche, y	
Mean (SD)	12.8 (1.9)
≤12	453 (46.7)
13	202 (20.8)
14	144 (14.8)
≥15	172 (17.7)
Unknown/missing	24
No. of pregnancies (gravida)	
Mean (SD)	3.1 (2.2)
0	116 (11.8)
1	117 (11.9)
2	197 (20.1)
≥3	551 (56.2)
Unknown/missing	14
No. of full-term pregnancies (para)	
Mean (SD)	2.4 (1.9)
0	147 (15.0)
1	158 (16.1)
2	273 (27.8)
≥3	404 (41.1)
Unknown/missing	13
Age at first pregnancy, parous women, y (n = 824)	
Mean (SD)	22.8 (5.3)
<20	260 (31.5)
20-24	289 (35.1)
25-29	185 (22.5)
≥30	90 (10.9)
TAH-BSO (n = 715)	
Yes	139 (16.3)
No	716 (83.7)
Unknown/missing	280
Menopausal status (n = 947)	
Premenopausal	558 (57.5)
Postmenopausal	413 (42.5)
Unknown/missing	48
Age at natural menopause (n = 236), y	
Mean (SD)	47.2 (5.6)
≤45	81 (34.3)
46-50	93 (39.4)
>50	62 (26.3)
Germline likely pathogenic/pathogenic variant status	
Positive	886 (86)
Negative	139 (14)
Estrogen receptor	
Positive	342 (59.5)
Negative	233 (40.5)
Unknown/missing	420

Abbreviations: BC, breast cancer; BMI, body mass index (calculated as weight in kilograms divided by height in meters squared); TAH-BSO, total abdominal hysterectomy with bilateral oophorectomy.

^a^ Participants chose among 12 categories; selecting multiple options was allowed. If the option Other was selected, they could write how they self-identified. For analysis purposes, categories were combined as Asian (includes Chinese or Other part of Asia), Black or Afro-Caribbean (includes African American, Afro-Caribbean, and Black), East Indian (includes India), 2 or more races (includes when participants selected ≥2 categories [eg, Chinese or other part of Asia, Afro-Caribbean, and White/Caucasian]), White or Caucasian (includes White/Caucasian and European).

**Table 2.** Demographic Characteristics of the Study Population Divided by Birth Group

Variable	Year of birth, No. (%)				*P* value
	<1950 (n = 177)	1950-1959 (n = 254)	1960-1969 (n = 330)	≥1970 (n = 234)	
Age at BC diagnosis, mean (SD), y	60.7 (9.4)	50.4 (7.6)	44.3 (4.9)	35.0 (5.0)^a^	<.001
Race^b^					
Asian^c^	0	0	0	3	<.001
Black or Afro-Caribbean	96 (83.5)	144 (88.3)	199 (77.1)	166 (82.6)	
East Indian	8 (7.0)	10 (6.1)	48 (18.6)	32 (15.9)	
≥ Races	1 (0.8)	3 (1.9)	6 (2.3)	2 (1)	
White or Caucasian	10 (8.7)	6 (3.7)	5 (2.0)	1 (0.5)	
Unknown/missing^c^	62	91	72	30	
BMI (n = 962)					
<25.0	42 (24.1)	52 (21.9)	71 (22.1)	78 (34.1)	.02
25.0-29.9	53 (30.5)	78 (32.8)	118 (36.8)	71 (31)	
≥30.0	79 (45.4)	108 (45.3)	132 (41.1)	80 (34.9)	
Family history of breast cancer (n = 986)					
Yes	93 (52.5)	136 (54.6)	163 (49.7)	105 (45.3)	.20
No	84 (47.5)	113 (45.4)	165 (50.3)	127 (54.7)	
Family history of ovarian cancer (n = 980)					
Yes	15 (8.6)	26 (10.5)	39 (12.0)	26 (11.2)	.71
No	159 (91.4)	222 (89.5)	287 (88)	206 (88.8)	
No. of siblings (n = 817)					
0	7 (4.2)	8 (3.5)	5 (2)	4 (2.4)	<.001
1-5	71 (42.5)	112 (48.5)	110 (44)	118 (69.8)	
>5	89 (53.3)	111 (48)	135 (54)	47 (27.8)	
Mean (SD)	6.6 (5.4)	5.8 (4.0)	6.1 (3.6)	4.5 (3.1)	<.001
Age at menarche, y (n = 971)					
≤12	57 (33.0)	116 (47.3)	148 (45.5)	132 (57.9)	<.001
13	36 (20.8)	51 (20.8)	68 (20.9)	47 (20.6)	
14	41 (23.7)	35 (14.3)	49 (15.1)	19 (8.3)	
≥15	39 (22.5)	43 (17.6)	60 (18.5)	30 (13.2)	
Mean (SD)	13.4 (1.9)	12.8 (1.9)	12.9 (1.8)	12.3 (1.7)	
No. of pregnancies (gravida) (n = 981)					
0	12 (6.8)	17 (6.8)	34 (10.5)	53 (22.8)	<.001
1	15 (8.6)	20 (8.0)	37 (11.4)	45 (19.4)	
2	28 (16)	56 (22.5)	70 (21.5)	43 (18.5)	
≥3	120 (68.6)	156 (62.7)	184 (56.6)	91 (39.2)	
Mean (SD)	4.0 (2.7)	3.5 (2.2)	2.9 (2.0)	2.1 (1.8)	
No. of full-term pregnancies (para) (n = 982)					
0	15 (8.6)	23 (9.2)	45 (13.9)	64 (27.6)	<.001
1	18 (10.3)	30 (12.0)	57 (17.5)	53 (22.8)	
2	41 (23.4)	73 (29.2)	94 (28.9)	65 (28.0)	
≥3	101 (57.7)	124 (49.6)	129 (39.7)	50 (21.6)	
Mean (SD)	3.5 (2.5)	2.8 (1.8)	2.2 (1.5)	1.6 (1.4)	
Age at 1st pregnancy, parous women, y (n = 824)					
<20	53 (33.3)	70 (31.5)	94 (33.7)	43 (26.2)	.12
20-24	58 (36.5)	92 (41.4)	83 (29.7)	56 (34.2)	
25-29	35 (22.0)	41 (18.5)	66 (23.7)	43 (26.2)	
≥30	13 (8.2)	19 (8.6)	36 (12.9)	22 (13.4)	
Mean (SD)	22.2 (5.1)	22.6 (5.2)	23.1 (5.8)	23.7 (5)	.06
TAH-BSO (n = 855)					
Yes	48 (32)	43 (20.8)	37 (13.2)	11 (5.1)	<.001
No	102 (68)	164 (79.2)	244 (86.8)	206 (94.9)	
Menopausal status (n = 947)					
Premenopausal	14 (8.3)	74 (30.3)	247 (75.5)	223 (96.5)	<.001
Postmenopausal	155 (91.7)	170 (69.7)	80 (24.5)	8 (3.5)	
Age at natural menopause, postmenopausal women, y (n = 236)					
≤45	20 (22.7)	30 (30.6)	28 (59.6)	3 (100)^a^	<.001
46-50	33 (37.5)	42 (42.9)	18 (38.3)	0^a^	
>50	35 (39.8)	26 (26.5)	1 (2.13)	0^a^	
Mean (SD)	49.1 (5.6)	47.6 (5)	43.3 (5)	41 (3.6)^a^	<.001
Germline likely pathogenic/pathogenic variant status					
Positive	17 (9.6)	30 (11.8)	52 (15.8)	40 (17.1)	.09
Negative	160 (90.4)	224 (88.2)	278 (84.2)	194 (82.9)	
Estrogen receptor					
Positive	55 (67.9)	73 (62.4)	117 (57.4)	97 (56.1)	.26
Negative	26 (32.1)	44 (37.6)	87 (42.6)	76 (43.9)	
Unknown/missing^c^	96	137	126	61	

Abbreviations: BC, breast cancer; BMI, body mass index (calculated as weight in kilograms divided by height in meters squared); TAH-BSO, total abdominal hysterectomy with bilateral oophorectomy.

^a^ The birth group that was born in or after 1970 was excluded from this analysis.

^b^ Participants chose among 12 categories; selecting multiple options was allowed. If the option Other was selected, they could write how they self-identified. For analysis purposes, categories were combined as Asian (includes Chinese or Other part of Asia) Black or Afro-Caribbean (includes African American, Afro-Caribbean, and Black) East Indian (includes India), Mixed (includes when participants selected ≥2 or more categories [eg, Chinese or other part of Asia], Afro-Caribbean, and White/Caucasian), White or Caucasian (includes White/Caucasian and European).

^c^ The category was excluded from the analysis.

Correlation analysis between age at BC diagnosis and other continuous variables showed that younger age at BC diagnosis correlated with younger age at menarche, fewer pregnancies, fewer FTPs, younger age at menopause, and older age at first pregnancy (correlation coefficient ρ or *P* value) (eTable 2 in Supplement 1). Mean (SD) age at BC diagnosis differed significantly between women who experienced menarche at or before age 12 (45 [10.5]) years compared with women who experienced menarche at 14 (49.9 [10.4]) years and at or after 15 (49.1 [11.2]) years (*P* < .001) (Figure, A). Women who were never pregnant had a lower mean (SD) age of BC (41.5 [11.4] years) compared with women with 2 pregnancies (46.1 [9.36] years) or those with 3 or more pregnancies (49.9 [11.2] years; *P* < .001) (Figure, B). Nulliparous women had BC diagnosed at a mean (SD) age of 42.1 (11.2) years compared with women with more than 2 FTPs who were diagnosed a mean of 7 years later (49.9 [10.6] years; *P* < .001) (Figure, C). There were no associations in age at BC diagnosis with age at first pregnancy (eFigure 1 in Supplement 1). Women who experienced natural menopause at or before age 45 years presented with a mean (SD) age at BC diagnosis of 52.6 (9.5) years, which differed from the age at BC diagnosis in women undergoing natural menopause between ages 46 and 50 years (57.1 [9.5] years; *P* < .001) and after 50 years (58.6 [8.3] years; *P* < .001) (Figure, D).

**Figure.**
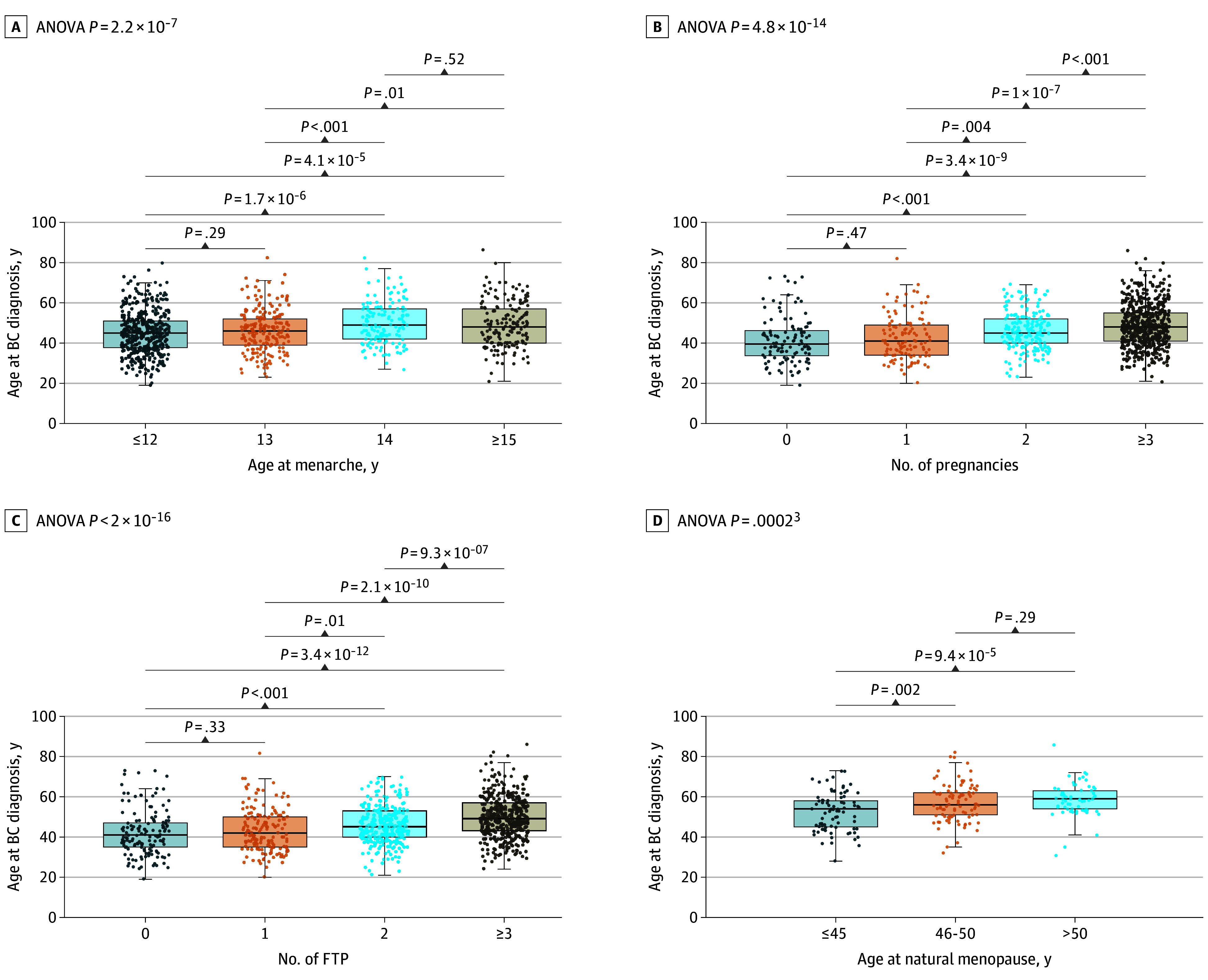
Comparison of Age at Breast Cancer (BC) Diagnosis Across Reproductive Factors A, Differences in women reporting menarche at or before age 12 (mean, 45) years, 13 (mean, 45.9) years, 14 (mean, 49.9) years, and at or after 15 (mean, 49.1) years. B, Differences among number of pregnancies, women with no pregnancies (mean age, 41.5 years), 1 pregnancy (mean age, 42.5 years), 2 pregnancies (mean age, 46.1 years), and 3 or more pregnancies (mean age, 48.7 years). C, Differences among number of full-term pregnancies (FTPs): nulliparous women (mean age, 42.1 years), 1 FTP (mean age, 43.4 years), 2 FTPs (mean age, 46 years) and 3 or more FTPs (mean age, 49.9 years). D, Differences among age of natural menopause: women experiencing menopause at or before 45 years (mean age, 52.6 years), between 46 and 50 years (mean age, 57.1 years), and after 50 years (mean age, 58.6 years). ANOVA indicates analysis of variance.

We explored age and BC diagnosis across each birth cohort, stratified by country. The distribution was uniform and similar (eFigure 2 in Supplement 1). The overall trend across all countries showed a decrease in the age at BC diagnosis as the birth cohort increased. Compared with women in the other countries, Bahamian women consistently presented at a younger mean age of BC diagnosis in each birth cohort.

Based on HBOC germline variant status, as expected, women with germline-positive variants had a lower mean age at BC diagnosis (40.6 years) compared with women with germline-negative variants (47.6 years), and family history differed by germline variant for BC (80.2% vs 45.7%; *P* < .001) or ovarian cancer (18.9% vs 9.6%; *P* = .001). There was a higher proportion of women who underwent TAH-BSO in the group with a positive germline variant (23.3%) compared with the group with a negative germline variant (15.3%; *P* = .04), as well as a higher proportion of ER-negative BC in women with a positive germline variant (53%) compared with those with none (38.9%; *P* = .03). There were no significant differences in the proportions or means for other variables, such as birth year, BMI, number of siblings, age at menarche, number of pregnancies, number of FTPs, age at first pregnancy, and age at natural menopause (Table 3).

**Table 3.** Characteristics of Study Population by Genetic Germline Variant Status

Variable	Germline likely pathogenic/pathogenic variant status, No. (%)		*P* value
**Positive**	**Negative**
Age at BC diagnosis, mean (SD), y	40.6 (9.0)	47.6 (10.7)	<.001
BMI (n = 962)			
<25.0	26 (19.9)	217 (26.1)	.09
25.0-29.9	50 (38.2)	270 (32.5)	
≥30.0	55 (42.0)	344 (41.4)	
Family history of breast cancer (n = 986)			
Yes	109 (80.2)	388 (45.7)	<.001
No	27 (19.9)	462 (54.4)	
Family history of ovarian cancer (n = 980)			
Yes	25 (18.9)	81 (9.6)	.001
No	107 (81.1)	767 (90.5)	
No. of siblings (n = 817)			
0	6 (4.4)	18 (2.7)	.40
1-5	72 (52.6)	339 (49.8)	
>5	59 (43.0)	323 (47.5)	
Mean (SD)	5.3 (3.5)	5.9 (4.2)	.07
Age at menarche, y (n = 971)			
≤12	64 (46.7)	389 (46.6)	.73
13	28 (20.4)	174 (20.9)	
14	24 (17.5)	120 (14.4)	
≥15	21 (15.3)	151 (18.1)	
Mean (SD)	12.8 (1.9)	12.8 (1.9)	.94
No. of pregnancies (gravida) (n = 981)			
0	15 (10.9)	101 (12)	.49
1	20 (14.5)	97 (11.5)	
2	32 (23.2)	165 (19.6)	
≥3	71 (51.4)	480 (56.9)	
Mean (SD)	2.9 (2.1)	3.1 (2.3)	.20
No. of full-term pregnancies (para) (n = 982)			
0	19 (13.8)	128 (15.2)	.35
1	29 (21)	129 (15.3)	
2	39 (28.2)	234 (27.7)	
≥3	51 (37)	353 (41.8)	
Mean (SD)	2.3 (1.7)	2.5 (1.9)	.22
Age at 1st pregnancy, parous women, y (n = 824)			
<20	37 (32.2)	223 (31.5)	.72
20-24	37 (32.2)	252 (35.5)	
25-29	30 (26.1)	155 (21.9)	
≥30	11 (9.5)	79 (11.1)	
Mean (SD)	23.3 (5.6)	22.9 (5.3)	.43
TAH-BSO (n = 855)			
Yes	24 (23.3)	115 (15.3)	.04
No	79 (76.7)	637 (84.7)	
Age at natural menopause, postmenopausal women, y (n = 236)			
≤45	10 (55.6)	71 (32.6)	.11
46-50	6 (33.3)	87 (39.9)	
>50	2 (11.1)	60 (27.5)	
Mean (SD)	44.2 (7.4)	47.5 (5.4)	.07
Estrogen receptor			
Positive	31 (47.0)	311 (61.1)	.03
Negative	35 (53.0)	198 (38.9)	
Unknown/missing	73	347	

Abbreviations: BC, breast cancer; BMI, body mass index (calculated as weight in kilograms divided by height in meters squared); TAH-BSO, total abdominal hysterectomy with bilateral oophorectomy.

Univariable and multivariable logistic regression models the likelihood of testing for a positive genetic variant in women across all birth groups overall and in The Bahamas are presented in eTable 3 in Supplement 1. As expected, women with a family history of BC or ovarian cancer were more likely to have a positive genetic germline variant (odds ratio [OR], 4.98; 95% CI, 3.13-7.92; *P* < .001). Furthermore, for every 1-year increase in age at first pregnancy, women had a 4% increased odds of receiving a diagnosis of ER-positive tumors (OR, 1.04; 95% CI, 1.01-1.08; *P* = .02) (eTable 4 in Supplement 1); other reproductive factors were not significant in the model testing the likelihood of testing for a positive germline variant or ER-positive tumors.

## Discussion

Several factors increase the risk of developing BC. Hormonal drivers and lifestyle changes noted with economic development and reproductive patterns all resulted in a decrease in the age at BC diagnosis.^21,22^ Our findings suggest a decreasing mean age in age at BC diagnosis in younger generations of Caribbean women. The youngest cohort was excluded from this analysis as women in this cohort were not old enough to develop postmenopausal BC. However, the fact that age at BC diagnosis decreased in the 3 oldest birth groups aligns with a broader global trend of increasing BC incidence rates across various age groups and regions from 1990 to 2017, as reported by Lima et al.^23^ Decreasing age at BC diagnosis is linked to shifts in reproductive factor patterns, such as age at menarche, age at first pregnancy, number of pregnancies, and parity, as seen in the oldest 3 birth cohorts of our study and other studies.^24^ In the Caribbean, this decrease in the age at BC diagnosis can likely be attributed to changing habits of women as their countries become increasingly westernized.^25,26^

In this study, age at menarche decreased with each generation and the proportion of women who experienced menarche at age 12 years or younger increased with each generation. These changes have also been observed in high-income countries, such as the UK, where age at menarche decreased from 13.4 years for women born between 1925 and 1929 to 12.8 years for women born between 1950 and 1955.^27^ The decrease in mean age at menarche seen in the cohort in our study is further evidence of the increase in the human development index and corresponding behaviors of individuals in Caribbean countries. Age at menarche and menopause are major determinants of endogenous hormone exposure, associated with cancer risk, but also with osteoporosis^28^ and heart disease.^29,30^ A Collaborative Group on Hormonal Factors in Breast Cancer published a meta-analysis of more than 100 000 women showing the risk of BC increasing by lengthening a woman’s reproductive years, where early menarche played a greater role than later menopause.^31^ In addition, younger age at menarche has been reported to increase the odds of triple-negative BC compared with luminal A (ER-positive/progesterone receptor–negative), particularly in African American women.^32^ Diet and lifestyle changes play an important role in the decrease in age at menarche and BC onset.^33^ With modern lifestyle changes, such as diet and sedentarism, the rate of obesity is increasing, which contributes to the risk of developing BC.^34^

Further evidence of the shift in dietary habits and exercise patterns is seen in the proportion of women with obesity (BMI≥30) decreasing over the decades in the cohort in this study. High BMI is a well-known risk factor for breast, ovarian, and many other cancers.^35^ Two reviews suggested that high BMI may either be a risk factor or a protective one, depending on menopausal status of women at the time of BC diagnosis. In premenopausal women, a high BMI is a protective factor,^36^ whereas in postmenopausal women, it is a risk factor.^37^ In one study examining postmenopausal BC in 28 409 women in the UK, researchers found a 5% increase in the incidence of cancer per 5-unit increase in BMI.^35^ Our study assessed BMI and menopausal status at the time of BC diagnosis, with most premenopausal and postmenopausal women having obesity; however, no further association was found between BMI and year of birth when stratified by menopausal status in our study. Although high BMI showed a positive correlation with the incidence of BC diagnosis, in the cohort in our study, it is not clear how BMI affects BC age at onset.

In our study, for each year of delayed pregnancy, there was an increased chance of ER-positive BC diagnosis, by 4%. The improvement of the human development index in the Caribbean experienced by more-recent birth cohorts in the 1970s, for example, may explain the shifts in other reproductive factors, such as age at first pregnancy. Age at first pregnancy has been reported as a major fertility risk factor for BC since the publication of findings in an international multicenter case-control study in 1970.^38^ Early menarche and later age at first pregnancy have been shown to synergistically contribute to an earlier and longer exposure to estrogen during the initial development of the breast gland, a crucial time that modulates the risk for BC.^26,39^ These factors likely contribute to the decrease seen in the age at BC diagnosis in younger generations.

The decreasing number of siblings seen in our study is indicative of transitioning of larger to smaller families. Family history is associated with an increased risk of BC; however, in the cohort in our study, there was no significant change in positive family history proportions when stratified by year of birth, suggesting that family history has been an unchanged risk factor across generations.

In our study, the proportion of nulliparous women significantly increased from older to younger generations. However, a study comparing parity and BC incidence showed that a change of increased incidence of BC occurred more than 3 decades before the secular decrease in parity, concluding that the increase in BC incidence cannot be attributed to decreasing changes in parity.^40^ Furthermore, women with a germline pathogenic variant had a lower mean age at BC diagnosis compared with women without a germline variant, a finding supported by previously published cohorts.^41,42^ When the cohorts were stratified by germline variant status, we found no significant difference in the number of siblings, age at menarche, number of pregnancies, number of FTPs, or age at first pregnancy. This finding is similar to that of a study by Pal et al,^43^ reporting that the fertility patterns experienced by *BRCA* variant carriers is similar to that of noncarriers. However, the percent of women who had a TAH-BSO was significantly different between both groups, with germline variant carriers having a higher percent of TAH-BSO, which can be attributed to the fact that most women with a positive germline variant had undergone risk-reducing surgery. Additionally, there is a higher percentage of ER-negative BC in germline variant carriers (women with positive germline variants); this finding is consistent with the literature reporting high rates of triple-negative BC in *BRCA1* variant carriers.^44^

Reproductive factors affecting BC have been studied in other populations. For example, in a separate cohort in Barbados,^45^ researchers found that older age at first pregnancy and nulliparity were significant risk factors for BC diagnosis, with an apparent dose effect of higher parity reducing risk, with similar results in Puerto Rico.^46^ However, the literature on social determinants for BC in these populations is very limited by study variability.^45^ In The Bahamas, BC is diagnosed in women at a younger age (mean, 42 years).^18,47^ Bahamian women were more likely to report a genetic germline variant, which explains the younger age seen in our Bahamian cohort. Reproductive factors did not appear to influence age at BC presentation in The Bahamas, as the genetic germline variant was strong enough to change BC presentation. As reported by Anders et al,^48^ younger age adjusted for all well-known prognostic factors is a powerful estimator for higher rates of recurrence risk and lower survival. The Bahamas showed the highest prevalence of *BRCA1* variants of any population studied to date by our group.^19^ In sub-Saharan Africa, changes in lifestyle and reproductive patterns have been influenced by improved nutrition and education.^49^ Furthermore, these changes have been associated with a shift in the incidence and age of onset of BC.^50,51^ Better nutrition leads to earlier menarche, while increased educational levels among women delay the age at first pregnancy.^52^ The Caribbean represents a transitioning region in which reproductive patterns and behaviors linked to BC are rapidly changing.

### Limitations

Our study is not void of limitations. Although our results show a decrease in the mean age at diagnosis of the first BC concurrent with changes in reproductive patterns, these trends may be associated with several other factors. First, we did not obtain a representative sample of all women unaffected by BC and our analyses focused on women with BC without a control group; therefore, certain well-known factors that affect BC incidence, such as age at first pregnancy, were not significant within cases of BC only. Second, since this was a cross-sectional and not a longitudinal study, we only surveyed women who survived long enough to participate in our study; therefore, the youngest cohort (born in or after 1970) was represented mainly by premenopausal women with BC. Furthermore, women with premenopausal BC may have experienced pregnancy after data were captured. Third, the main sources of exogenous estrogen exposure, such as hormone replacement therapy and oral contraceptive use, can increase the risk of BC,^53^ and these variables were not captured in this study. Fourth, a limited number of participants had immunohistochemistry test results available, so BC molecular subtypes and reproductive patterns could not be robustly assessed. Fifth, participants in the study included women from 7 countries of the Caribbean; the observed changes in reproductive patterns may differ in other populations.

## Conclusions

The data from this cross-sectional study suggest that The Bahamas, Barbados, Cayman Islands, Dominica, Haiti, Jamaica, and Trinidad and Tobago have undergone changes in reproductive patterns, specifically a decrease in the number of siblings, age at menarche, number of pregnancies, number of FTPs, and age at menopause, within the 4 age cohorts studied. These changes provide insight into risk factor patterns for BC incidence associated with younger age at BC diagnosis. As Caribbean countries experience this transition, interventions targeting other modifiable risk factors for BC, such as dietary intake of fruits and vegetables, increased physical activity, weight loss, and decreased alcohol consumption, are needed to compensate for the shift in reproductive patterns.

## Notes

Supplement 1. **eTable 1.** Stratification of BMI by Menopausal Status **eTable 2.** Spearman Correlation Between Age at BC Diagnosis and Other Variables **eFigure 1.** No Differences Seen Comparing Age at BC Diagnosis in Women Reporting Age at First Pregnancy Before 20y (Mean 48.4y), Between 20-24y (Mean 48.6y), Between 25-29y (mean 47.9y), and at or After 30y (Mean 48.7y) **eFigure 2.** Mean Age at Breast Cancer Diagnosis by Decade of Birth and 95% Confidence Interval Bars Are Shown **eTable 3.** Logistic Regression Testing the Likelihood of a Positive Germline Pathogenic/Likely Pathogenic Variant **eTable 4.** Logistic Regression Testing the Likelihood of ER-Positive BC Diagnosis

Supplement 2.
Data Sharing Statement
