# Supplementary material for: Reproductive Risk Factor Patterns in Caribbean Women With Breast Cancer Across 4 Generations
Source: JAMA Netw Open. 2024 Oct 8;7(10):e2438091. doi: 10.1001/jamanetworkopen.2024.38091 (PMC11581535; doi:10.1001/jamanetworkopen.2024.38091)
Supplement: Supplement 1. — eTable 1. Stratification of BMI by Menopausal Status eTable 2. Spearman Correlation Between Age at BC Diagnosis and Other Variables eFigure 1. No Differences Seen Comparing Age at BC Diagnosis in Women Reporting Age at First Pregnancy Before 20y (Mean 48.4y), Between 20-24y (Mean 48.6y), Between 25-29y (mean 47.9y), and at or After 30y (Mean 48.7y) eFigure 2. Mean Age at Breast Cancer Diagnosis by Decade of Birth and 95% Confidence Interval Bars Are Shown eTable 3. Logistic Regression Testing the Likelihood of a Positive Germline Pathogenic/Likely Pathogenic Variant eTable 4. Logistic Regression Testing the Likelihood of ER-Positive BC Diagnosis [file jamanetwopen-e2438091-s001.pdf]

Supplementary Online Content

Sanchez-Covarrubias AP, Chery MJ, Barreto-Coehlo P, et al. Reproductive risk factor patterns in Caribbean women with breast cancer across 4 generations. *JAMA Netw Open*. 2024;7(10):e2438091. doi:10.1001/jamanetworkopen.2024.38091

eTable 1. Stratification of BMI by Menopausal Status

eTable 2. Spearman Correlation Between Age at BC Diagnosis and Other Variables

eFigure 1. No Differences Seen Comparing Age at BC Diagnosis in Women Reporting Age at First Pregnancy Before 20y (Mean 48.4y), Between 20-24y (Mean 48.6y), Between 25-29y (mean 47.9y), and At or After 30y (Mean 48.7y)

eFigure 2. Mean Age at Breast Cancer Diagnosis by Decade of Birth and 95% Confidence Interval Bars Are Shown

eTable 3. Logistic Regression Testing the Likelihood of a Positive Germline Pathogenic/Likely Pathogenic Variant

eTable 4. Logistic Regression Testing the Likelihood of ER-Positive BC Diagnosis

This supplementary material has been provided by the authors to give readers additional information about their work.

© 2024 Sanchez-Covarrubias AP et al. *JAMA Network Open.*

eTable 1. Stratification of BMI by menopausal status

Variable	Total subjects	Year of Birth				p-value
		**< 1950 (n=177)**	**1950 – 1959 (n=254)**	**1960 – 1969 (n=330)**	**≥ 1970 (n=234)**	
**Pre-menopausal (N=537)**						
**BMI, kg/m^2**						0.3547
< 25, No. (%)	152 (28.3)	5 (35.7)	19 (28.8)	55 (23.1)	73 (33.3)	
25 – 29.9, No. (%)	183 (34.1)	4 (28.6)	24 (36.3)	85 (35.7)	70 (32)	
≥30, No. (%)	202 (37.6)	5 (35.7)	23 (34.9)	98 (41.2)	76 (34.7)	
**Post-menopausal (N=406)**						
**BMI, kg/m^2**						0.2235
< 25, No. (%)	84 (20.7)	33 (21.6)	32 (19.4)	15 (18.8)	4 (50)	
25 – 29.9, No. (%)	132 (32.5)	48 (31.4)	50 (30.3)	33 (41.2)	1 (12.5)	
≥30, No. (%)	190 (46.8)	72 (47.1)	83 (50.3)	32 (40)	3 (37.5)	

© 2024 Sanchez-Covarrubias AP et al. *JAMA Network Open.*

eTable 2. Spearman correlation between age at BC diagnosis and other variables

**Variables**	**Age at Breast Cancer diagnosis**
Age of Menarche	n = 970<br>ρ = 0.170, p <.001
Number of Pregnancies (Gravida)	n = 980<br>ρ = 0.311, p<.001
Number of FTP (Para)	n = 981<br>ρ = 0.332, p<.001
Age of 1st pregnancy	n = 850<br>ρ = -0.098, p=0.004
Age of Natural Menopause	n = 236<br>ρ = 0.340, p<.001

ρ: Spearman's rank correlation coefficient

© 2024 Sanchez-Covarrubias AP et al. *JAMA Network Open.*

eFigure 1. No differences seen comparing age at BC diagnosis in women reporting age at first pregnancy before 20y (mean 48.4y), between 20-24y (mean 48.6y), between 25-29y (mean 47.9y) and at or after 30y (mean 48.7y).

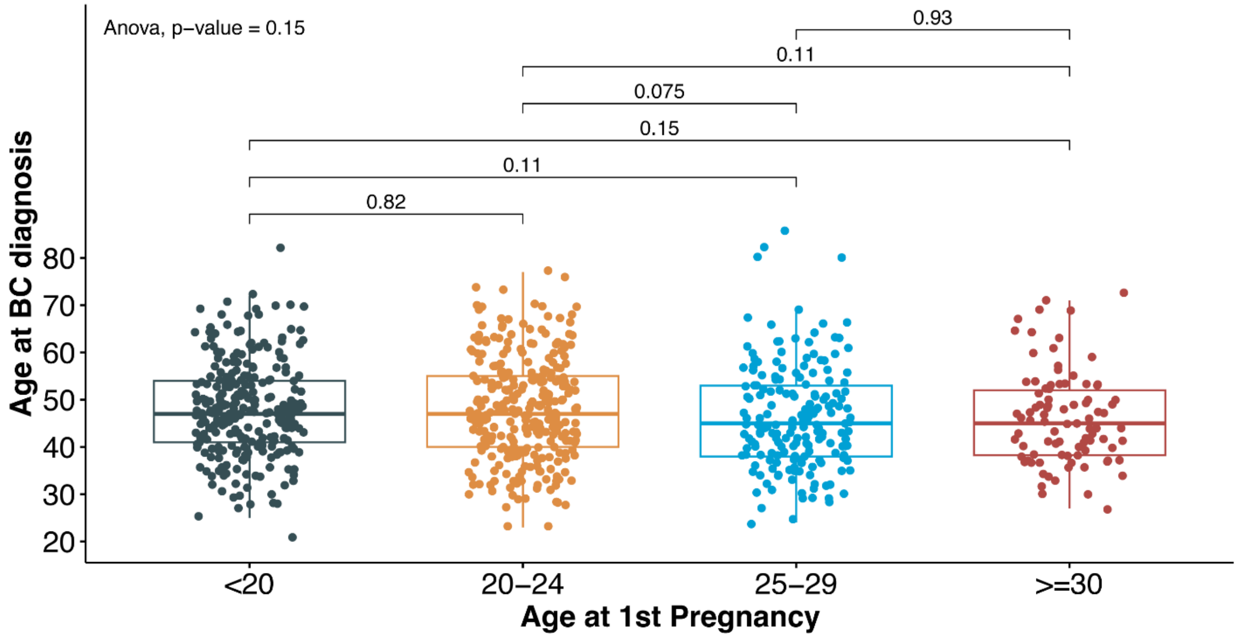

© 2024 Sanchez-Covarrubias AP et al. *JAMA Network Open.*

eFigure 2. Mean age at breast cancer diagnosis by decade of birth and 95% confidence interval bars are shown. There was significant difference between mean age of BC diagnosis across different decades of birth for each island in which the study was conducted.

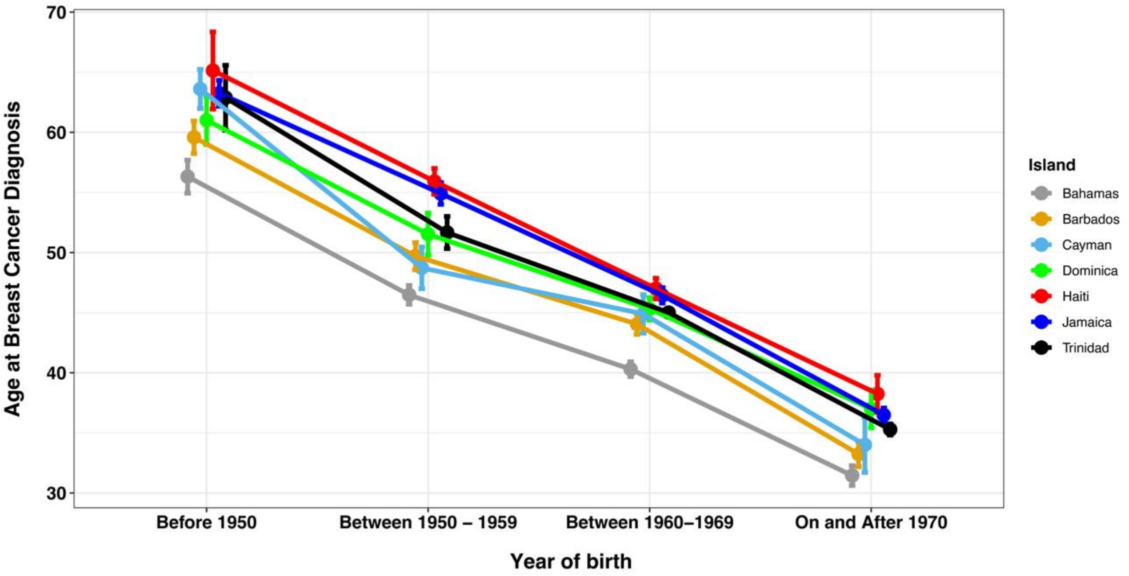

© 2024 Sanchez-Covarrubias AP et al. *JAMA Network Open.*

eTable 3. Logistic regression testing the likelihood of a Positive Germline Pathogenic/likely pathogenic Variant.

	**Univariable Analysis**			**Multivariable Analysis**		
	Odds Ratio (OR)	95% CI	p-value	Odds Ratio (OR)	95% CI	p-value
**ALL SUBJECTS**						
**Year of birth**						
< 1950	reference			reference		
1950 – 1959	1.26	(0.67 – 2.36)	0.470	1.12	(0.59 – 2.15)	0.730
1960 – 1969	1.76	(0.99 – 3.15)	0.056	1.78	(0.98 – 3.24)	0.057
≥ 1970	1.94	(1.06 – 3.55)	0.032	2.15	(1.15 – 3.99)	0.016
**FH of Breast or Ovarian cancer**						
No	reference			reference		
Yes	4.78	(3.01 – 7.57)	<.001	4.98	(3.13 – 7.92)	<.001
**THE BAHAMAS**						
**Year of birth**						
< 1950	reference			reference		
1950 – 1959	1.38	(0.57 – 3.31)	0.475	1.19	(0.47 – 2.97)	0.716
1960 – 1969	2.78	(1.17 – 6.61)	0.021	2.80	(1.13 – 6.95)	0.026
≥ 1970	4.12	(1.51 – 11.27)	0.006	4.06	(1.41 – 11.66)	0.009
**FH of Breast or Ovarian cancer**						
No	reference			reference		
Yes	5.37	(2.42 – 11.95)	<.001	5.59	(2.47 – 12.63)	<.001

© 2024 Sanchez-Covarrubias AP et al. *JAMA Network Open.*

eTable 4. Logistic regression testing the likelihood of ER-positive BC diagnosis.

	**Univariable Analysis**		
	Odds Ratio (OR)	95% CI	p-value
**Year of birth**			
< 1950	Reference		
1950 – 1959	0.78	(0.43 – 1.43)	0.426
1960 – 1969	0.64	(0.37 – 1.09)	0.102
≥ 1970	0.60	(0.35 – 1.05)	0.074
**Age at 1st Pregnancy**			
Per year increased	1.04	(1.01 – 1.08)	0.018
< 22y	Reference		
≥ 22y	1.70	(1.18 – 2.44)	0.004

© 2024 Sanchez-Covarrubias AP et al. *JAMA Network Open.*
