# Supplementary material for: Reproductive Risk Factor Patterns in Caribbean Women With Breast Cancer Across 4 Generations
Source: JAMA Netw Open. 2024 Oct 8;7(10):e2438091. doi: 10.1001/jamanetworkopen.2024.38091 (PMC11581535; doi:10.1001/jamanetworkopen.2024.38091)
Supplement: Supplement 2. — Data Sharing Statement [file jamanetwopen-e2438091-s002.pdf]

Data Sharing Statement

Sanchez-Covarrubias. Reproductive Risk Factor Patterns in Caribbean Women With Breast Cancer Across 4 Generations. *JAMA Netw Open*. Published October 08, 2024. doi:10.1001/jamanetworkopen.2024.38091

Data
Data available: No
